# Inter-Species Pharmacokinetic Modeling and Scaling for Drug Repurposing of Pyronaridine and Artesunate

**DOI:** 10.3390/ijms25136998

**Published:** 2024-06-26

**Authors:** Dong Wook Kang, Ju Hee Kim, Kyung Min Kim, Seok-jin Cho, Go-Wun Choi, Hea-Young Cho

**Affiliations:** College of Pharmacy, CHA University, 335 Pangyo-ro, Bundang-gu, Seongnam-si 13488, Gyeonggi-do, Republic of Korea; dongwk203@gmail.com (D.W.K.); juheekim907@gmail.com (J.H.K.); mini805mini@gmail.com (K.M.K.); sj20219106@gmail.com (S.-j.C.); gwchoi@chauniv.ac.kr (G.-W.C.)

**Keywords:** pharmacokinetics, inter-species scaling, allometric scaling, drug repurposing, pyronaridine, artesunate

## Abstract

Even though several new targets (mostly viral infection) for drug repurposing of pyronaridine and artesunate have recently emerged in vitro and in vivo, inter-species pharmacokinetic (PK) data that can extend nonclinical efficacy to humans has not been reported over 30 years of usage. Since extrapolation of animal PK data to those of humans is essential to predict clinical outcomes for drug repurposing, this study aimed to investigate inter-species PK differences in three animal species (hamster, rat, and dog) and to support clinical translation of a fixed-dose combination of pyronaridine and artesunate. PK parameters (e.g., steady-state volume of distribution (V_ss_), clearance (CL), area under the concentration-time curve (AUC), mean residence time (MRT), etc.) of pyronaridine, artesunate, and dihydroartemisinin (an active metabolite of artesunate) were determined by non-compartmental analysis. In addition, one- or two-compartment PK modeling was performed to support inter-species scaling. The PK models appropriately described the blood concentrations of pyronaridine, artesunate, and dihydroartemisinin in all animal species, and the estimated PK parameters in three species were integrated for inter-species allometric scaling to predict human PKs. The simple allometric equation (*Y* = *a* × *W^b^*) well explained the relationship between PK parameters and the actual body weight of animal species. The results from the study could be used as a basis for drug repurposing and support determining the effective dosage regimen for new indications based on in vitro/in vivo efficacy data and predicted human PKs in initial clinical trials.

## 1. Introduction

Traditionally, pyronaridine has been used as a single agent or artemisinin-based combination therapy for malaria treatment [1]. The activity of pyronaridine is highly potent against *Plasmodium falciparum*, including strains resistant to other antimalarials [2]. In erythrocytes, malaria parasites digest hemoglobin for replication and neutralize the toxicity of free heme (released by hemoglobin digestion) by converting free heme into hemozoin [3]. Pyronaridine blocks the formation of hemozoin and facilitates the accumulation of toxic heme in the digestive vacuole of malaria parasites; thus, the growth and replication of parasites are inhibited [4]. Currently, pyronaridine has been approved only by the European Medicines Agency (EMA) under the brand name Pyramax^®^ (Seoul, Republic of Korea) for the indication of the treatment of malaria infection as a fixed-dose combination with artesunate [5]. The World Health Organization (WHO) recommends artemisinin-based combination therapy for malaria treatment [6]. Short-acting artemisinin derivatives (artesunate, artemether, dihydroartemisinin, etc.) and long-acting antimalarial agents (pyronaridine, mefloquine, etc.) that have a different mechanism of action have been combined in artemisinin-based combination therapy [7]. Artesunate was approved by the Food and Drug Administration (FDA) in the United States under the brand name Amivas^®^ (Wilmington, DE, USA) for treating severe malaria [8]. Although artesunate has antimalarial effects as a monotherapy, the 3:1 ratio of the pyronaridine:artesunate combination significantly lowers the ED_90_ of pyronaridine or artesunate administered alone [9,10]. Artesunate is rapidly and almost wholly hydrolyzed to dihydroartemisinin, the principal active metabolite of artesunate [9,11]. Artesunate undergoes biotransformation primarily in the liver, where it is metabolized to dihydroartemisinin (DHA) by cytochrome 2A6 and blood esterase [12]. Human liver microsomal incubations of DHA revealed the presence of only DHA-glucuronide as a metabolite (catalyzed by UGT1A9 and UGT2B7), while α-DHA-β-glucuronide (α-DHA-G) and varying amounts of the tetrahydrofuran isomer of α-DHA-G were identified in urine samples from patients, with DHA itself detected only in trace amounts [5,12]. Dihydroartemisinin is the active metabolite of all artemisinin-like compounds and has antimalarial effects with low toxicity [13]. The mechanism of action for artesunate and dihydroartemisinin includes heme polymerization, alkylating malarial proteins, and ROS generation, which results in oxidative damage to malaria parasites [14,15].

Recent studies have demonstrated pyronaridine has immune-modulatory activity in immune cells as well as anti-cancer activity via anti-mitochondrial activity, altering cell cycle progression, and intercalating DNA [16,17,18]. These studies have shown that pyronaridine may be a promising candidate for antiparasitic, anticancer, antiviral, etc. [19,20,21]. Artesunate and dihydroartemisinin have also been studied for their usage in non-malarial indications. Artesunate is well-tolerated and has shown high efficacy in anti-parasitic, anti-viral, anti-tumor, and anti-inflammatory effects [22,23,24]. Also, dihydroartemisinin regulates immune system function and inhibits tumor growth and inflammation, in addition to having antimalarial effects [13]. Based on the evidence from nonclinical studies, several attempts at drug repurposing of pyronaridine and artesunate have been ongoing. As a monotherapy, there is a ton of research for drug repurposing of pyronaridine for the treatment of breast cancer, *Echinococcus granulosus* infection, Ebola/Marburg virus infection, and severe acute respiratory syndrome coronavirus 2 (SARS-CoV-2) infection [18,19,20,25]. Also, new indications for treating metastatic renal cell carcinoma, hepatocellular carcinoma, hepatitis E virus, depression prevention, and ovarian cancer have been studied for artesunate [26,27,28,29,30,31]. In addition to the monotherapy, research for the approved drug, Pyramax^®^ (3:1 fixed-dose combination of pyronaridine:artenusate), has been conducted to uncover new indications of Ebola virus, influenza, and SARS-CoV-2 disease for drug repurposing [32,33,34].

Drug repurposing of approved drugs is generally considered a time-saving, low-cost, and minimum-risk strategy to identify new therapeutic usage [35]. Since approved drugs have already established their safety in animals and humans, the drugs could be approved by evaluating their efficacy for the new indications in nonclinical studies and clinical trials [36]. In our previous study, the pharmacokinetics of pyronaridine and artesunate were evaluated in hamsters to support in vivo activity for drug repurposing to non-malarial effects [37]. Although new molecular targets of pyronaridine and artesunate (including dihydroartemisinin, its active metabolite) have continually emerged in recent years, PK data that could support those new efficacy data are insufficient. To support drug repurposing, this study described the inter-species PK differences of pyronaridine, artesunate, and dihydroartemisinin in three animal species (hamsters, rats, and dogs) and predicted the clinical PK by inter-species scaling of PK parameters. The PK parameters were estimated using non-compartmental analysis (NCA) and compartment PK modeling. Integrating the PK parameters in animal species, clinical PK parameters were predicted using the allometric scaling and compared with the reported human PK parameters.

## 2. Results

### 2.1. Pharmacokinetics of Pyronaridine, Artesunate, and Dihydroartemisinin in Hamsters, Rats, and Dogs

The pharmacokinetics of pyronaridine were evaluated in hamsters, rats, and dogs. Because a high blood-to-plasma ratio (4.9–17.8) and distribution to red blood cells have been reported for pyronaridine, whole blood was selected as the matrix for bioanalytical quantification [38]. The PK profile of pyronaridine for each species is shown in Figure S1. The corresponding estimated PK parameters from NCA for each species are summarized in Table S1. In rats, 60 mg/kg of pyronaridine tetraphosphate was orally administered once or once a day for three days. From the single-dosing group (Figure S1a, left), the V_ss_/F and CL/F were estimated to be 125.02 ± 21.63 L/kg and 2.79 ± 0.21 L/h/kg in rats. The T_max_, t_1/2_, and MRT were estimated to be 3.83 ± 3.43, 37.43 ± 6.61, and 45.01 ± 8.60 h, respectively. In dogs, 90 mg of pyronaridine tetraphosphate was orally administered once (Figure S1a, right). The V_ss_/F and CL/F were estimated to be 28.40 ± 5.55 L/kg and 0.64 ± 0.13 L/h/kg. The T_max_, t_1/2_, and MRT were estimated to be 1.67 ± 1.09, 38.44 ± 7.06, and 46.19 ± 11.47 h.

The standard deviation could not be calculated in hamsters since naïve-pooled analysis (pooling sparse data from several individuals) was used to evaluate PKs. In the multiple-dosing group (180 mg/kg or 360 mg/kg for three days) in hamsters (Figure S1b, left), the t_1/2_ and MRT were estimated to be 14.05–13.97 h and 20.27–20.15 h for the low-to-high dose group, respectively. The accumulation index was calculated to be 2.37 for the low-dose group and 1.66 for the high-dose group in hamsters. The V_ss_/F and CL/F were estimated to be 36.38–48.66 L/kg and 1.80–2.41 L/h/kg for the low-to-high dose group. In the multiple-dosing group (60 mg/kg for three days) in rats (Figure S1b, right), the t_1/2_ and MRT were estimated to be 42.27 ± 2.99 h and 60.99 ± 4.31 h. The V_ss_/F and CL/F were estimated to be 141.30 ± 18.66 L/kg and 2.33 ± 0.36 L/h/kg. The accumulation index was calculated to be 3.04 ± 0.45 in rats.

The pharmacokinetics of artesunate and dihydroartemisinin were evaluated in hamsters and rats. Since the blood-to-plasma ratio was reported to be 0.75 for both artesunate and dihydroartemisinin, blood concentrations were calculated by multiplying plasma concentrations by 0.75 [39]. The PK profiles of artesunate and dihydroartemisinin for each species are shown in Figure S2. The corresponding estimated PK parameters from NCA for each species are summarized in Table S2. Hamsters received a low dose (60 mg/kg) or a high dose (120 mg/kg) of daily oral artesunate for three consecutive days, and blood concentrations for artesunate and dihydroartemisinin were simultaneously quantified (Figure S2b). The t_1/2_ and MRT of artesunate were estimated to be 1.18–1.39 h and 1.70–2.00 h for the low-to-high dose group, respectively. The accumulation index of artesunate was calculated to be 0.11 for the low-dose group and 0.15 for the high-dose group. The V_ss_/F and CL/F of artesunate were estimated to be 11,915.76–19,674.11 L/kg and 7009.38–9830.19 L/h/kg for the low-to-high dose group. The t_1/2_ and MRT of dihydroartemisinin were estimated to be 0.31–0.33 h and 0.44–0.47 h for the low-to-high dose group, respectively. The accumulation index of dihydroartemisinin was calculated to be 0.17 for the low-dose group and 0.09 for the high-dose group.

In rats, blood concentrations for artesunate and dihydroartemisinin were simultaneously quantified after receiving 20 mg/kg of oral artesunate once or once a day for three days. From the single-dosing group (Figure S2a), the V_ss_/F and CL/F of artesunate were estimated to be 231.89 ± 116.35 L/kg and 547.08 ± 132.41 L/h/kg. The T_max_, t_1/2_, and MRT were estimated to be 0.14 ± 0.04, 0.17 ± 0.07, and 0.44 ± 0.23 h for artesunate and 0.28 ± 0.09, 0.23 ± 0.08, and 0.52 ± 0.11 h for dihydroartemisinin. From the multiple-dosing group (Figure S2c), the V_ss_/F and CL/F of artesunate were estimated to be 485.07 ± 189.31 L/kg and 1008.34 ± 593.65 L/h/kg. The t_1/2_ and MRT were estimated to be 0.43 ± 0.25 and 0.62 ± 0.36 h for artesunate and 0.31 ± 0.16 and 0.45 ± 0.23 h for dihydroartemisinin. The accumulation index of artesunate and dihydroartemisinin was calculated to be 0.13 ± 0.06 and 0.09 ± 0.08 in rats. The AUC_Day 1_ and AUC_Day 3_ of artesunate were 506.74 ± 170.31 and 69.01 ± 39.58, respectively, and showed a significant difference (*p* < 0.01). Also, the AUC_Day 1_ and AUC_Day 3_ of dihydroartemisinin were 943.37 ± 646.81 and 79.46 ± 69.28, respectively, and showed a significant difference (*p* < 0.01).

### 2.2. Compartment PK Modeling in Hamsters, Rats, and Dogs

**Figure 1:**
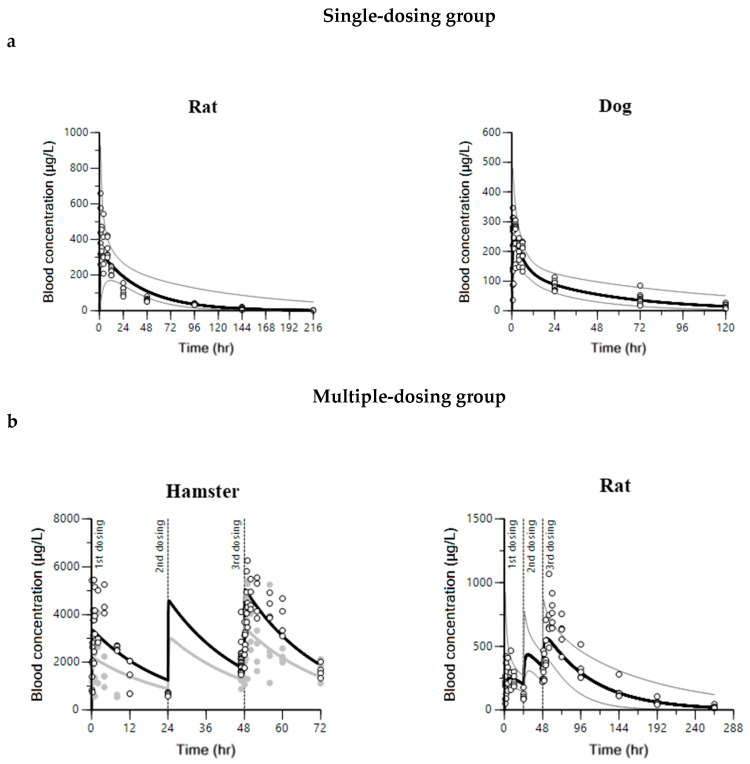
Model predictions and observations for blood concentrations of pyronaridine. (**a**) Single-dose PK profile for rats and dogs. (**b**) Multiple-dose PK profiles for hamsters and rats. Circles and lines are individual and average blood concentrations, respectively. The gray solid line represents the 2.5th–97.5th percentile range. In the hamster PK profile, closed circles with gray solid lines represent the low-dose group, and open circles with black solid lines represent the high-dose group.

**Figure 2:**
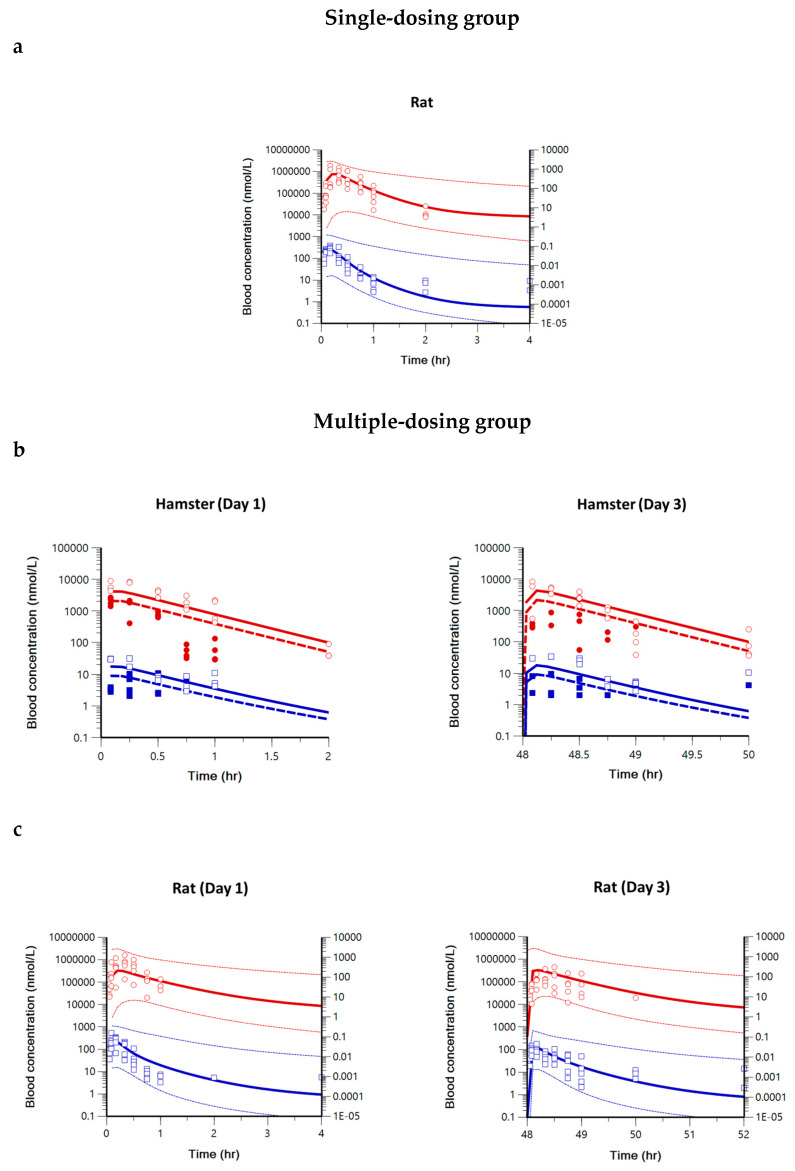
Model predictions and observations for blood concentrations of artesunate and dihydroartemisinin. (**a**) Single-dose PK profile for rats. (**b**) Multiple-dose PK profile for hamsters. (**c**) Multiple-dose PK profile for rats. Blue squares with blue lines represent artesunate (left *y*-axis), and red circles with red lines represent dihydroartemisinin (right *y*-axis), respectively. The dotted lines represent the 2.5th–97.5th percentile range. In the hamster PK profile, closed squares and circles with dashed lines represent the low-dose group, and open squares and circles with solid lines represent the high-dose group.

The non-compartmental PK parameters were used as initial estimates for compartment PK modeling of pyronaridine, artesunate, and dihydroartemisinin. A one- or two-compartment PK model was used to fit the observed blood concentrations in hamsters, rats, and dogs. The PK modeling process for pyronaridine, artesunate, and dihydroartemisinin is summarized in Tables S3 and S4. The one-compartment model described the blood concentrations of pyronaridine in hamsters and rats well, whereas the two-compartment model described those in dogs well. A lag time was not incorporated into the model parameter since the lag time was estimated to be zero in NCA. The blood concentrations of artesunate and dihydroartemisinin were well-described by the parent-metabolite model with auto-induction elimination in hamsters and rats. The combined model of one- (artesunate) and one- (dihydroartemisinin) compartment was established for hamsters, and the combined model of two- (artesunate) and one- (dihydroartemisinin) compartment was established for rats. To verify the performance of the PK models in each species, basic goodness-of-fit (GOF) plots were evaluated (Figures S3 and S4). The GOF plots showed no visible trends and suggested reasonable agreements between observations and model predictions. The blood concentrations for observations and model predictions in each species are shown in Figure 1 (pyronaridine) and Figure 2 (artesunate and dihydroartemisinin).

The estimated PK parameters from the developed model in all species are summarized in Table 1. The k_a_ for pyronaridine in hamsters, rats, and dogs was estimated to be 11.62, 1.67, and 2.46, respectively. The k_a_ for artesunate was estimated to be 2.08^−1^ in hamsters and 1.54 h^−1^ in rats, respectively. In compartmental PK analysis, the V_ss_ is described as V_c_ + V_p_, where V_c_ and V_p_ are the volumes of a central and peripheral compartment, respectively [40]. In other words, the V_ss_ is expressed as V_1_ (in the one-compartment model) or as V_1_ + V_2_ (in the two-compartment model) [41]. Thus, using the PK model estimates, the Vss/F of pyronaridine for hamsters, rats, and dogs was calculated to be 53.32, 110.00, and 32.68 L/kg. The elimination clearance (CL/F) of pyronaridine for hamsters, rats, and dogs was estimated to be 2.15, 2.18, and 0.68 L/h/kg, respectively. From the parent-metabolite PK model, the V_ss_/F of artesunate was calculated to be 1571.71 and 514.08 L/kg in hamsters and rats, and the V_m_/(F∙F_m_) of dihydroartemisinin was estimated to be 0.02 and 0.16 L/kg in hamsters and rats, respectively. The CL/F of artesunate was estimated to be 3555.94 and 13.33 L/kg in hamsters and rats, and the CL_m_/(F∙F_m_) of dihydroartemisinin was estimated to be 117.68 and 237.25 L/kg in hamsters and rats, respectively. These PK parameters in three species were used to extrapolate and predict PK parameters in humans.

### 2.3. Prediction of Human PK Parameters Using Allometric Scaling

**Figure 3:**
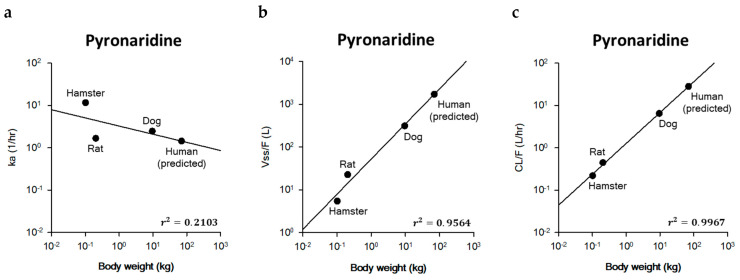
Relationship between (**a**) the absorption rate constant (k_a_), (**b**) the steady-state volume of distribution (V_ss_/F), and (**c**) the clearance (CL/F) of pyronaridine and body weight in hamsters, rats, dogs, and humans. The allometric equations are log *Y* = −0.1928 × log *W* + 0.5146 for k_a_, log *Y* = 0.8220 × log *W* + 1.7193 for V_ss_/F, and log *Y* = 0.7296 × log *W* + 0.1048 for CL/F, respectively.

The PK parameters estimated by the model fitting in hamsters, rats, and dogs were used for the inter-species extrapolation to predict human parameters of pyronaridine. The k_a_ of 11.62 h^−1^ (hamster), 1.67 h^−1^ (rat), and 2.46 h^−1^ (dog) was used for inter-species scaling. For the extrapolation, the units of V_ss_/F and CL/F were modified from L/kg to L and from L/h/kg to L/h, respectively, using the actual body weight of animals. The V_ss_/F in hamsters, rats, and dogs were calculated to be 5.44, 22.57, and 311.18 L, respectively. The CL/F in hamsters, rats, and dogs were estimated to be 0.22, 0.45, and 6.48 L/h, respectively. The body weights of hamsters, rats, and dogs used for allometric scaling were 0.10, 0.21, and 9.52 kg, respectively. The exponents, coefficients, and correlation coefficients for k_a_, V_ss_/F, and CL/F were estimated using the equation for simple allometric scaling. The human PK parameters were predicted using a simple allometric equation. The predicted parameters were 1.44 h^−1^ for k_a_, 1721.75 L (=24.60 L/kg) for V_ss_/F, and 28.25 L/h (=0.40 L/h/kg) for CL/F in a 70 kg human. The coefficient of determination (r^2^) was estimated to be 0.2103 for k_a_, 0.9564 for V_ss_/F, and 0.9967 for CL/F, respectively. The relationship between the PK parameters and body weights across the species is shown in Figure 3.

**Figure 4:**
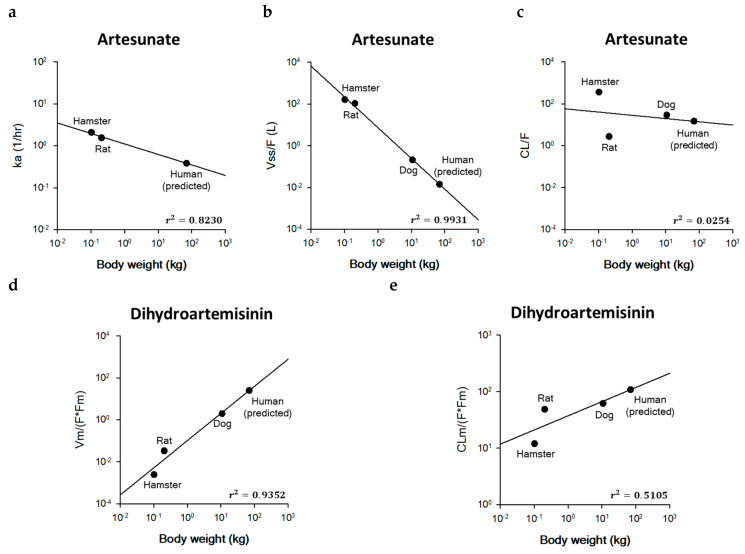
Relationship between (**a**) the absorption rate constant (k_a_), (**b**) the steady-state volume of distribution (V_ss_/F), and (**c**) the clearance (CL/F) of artesunate and body weight in hamsters, rats, dogs, and humans. (**d**) the volume of distribution [V_m_/(F·F_m_)] and (**e**) the clearance [CL_m_/(F·F_m_)] for dihydroartemisinin. The allometric equations for artesunate are log *Y* = −0.250 × log *W* + 0.0428 for k_a_, log *Y* = −1.4725 × log *W* + 0.8671 for V_ss_/F, and log *Y* = −0.1547 × log *W* + 1.4524 for CL/F, respectively. The allometric equations for dihydroartemisinin are log *Y* = 1.2933 × log *W* − 0.9876 for V_m_/(F·F_m_), and log *Y* = 0.2509 × log *W* + 1.5725 for CL_m_/(F·F_m_), respectively.

The estimated PK parameters from the model fitting in hamsters and rats were used for inter-species extrapolation to predict human parameters of artesunate and dihydroartemisinin. Similar to pyronaridine, the units of the volume of distribution and clearance were modified by multiplying the actual body weight of animals. In the case of artesunate, the V_ss_/F in hamsters and rats was calculated to be 160.33 and 105.48 L, and the CL/F in hamsters and rats was calculated to be 362.74 and 2.73 L/h. In the case of artesunate, the V_ss_/F and CL/F were estimated to be 160.33 L and 362.74 L/h in hamsters and 105.48 L and 2.73 L/h in rats. In the case of dihydroartemisinin, the V_m_/(F·F_m_) and CL_m_/(F·F_m_) were calculated to be 0.002 L and 12.00 L/h in hamsters and 0.033 L and 48.68 L/h in rats. In addition, the PK parameters of artesunate and dihydroartemisinin in dogs were taken from the literature, and only one study has reported the PK parameters of artesunate and dihydroartemisinin in dogs after intravenous (IV) injection of artesunate. In the dog PK study, the PK parameters of artesunate and dihydroartemisinin were reported to be 0.21 L for V_ss_/F, 28.86 L/h for CL/F, 1.96 L/kg for V_m_/(F·F_m_), and 61.54 L/h/kg for CL_m_/(F·F_m_) [42]. Since IV injection is an extravascular route, the absorption rate constant (k_a_) of artesunate cannot be estimated in dogs. Thus, after fixing the exponent of first-order rate constants (i.e., k_a_) to −0.25 following the literature, only the intercept for the k_a_ of artesunate was estimated by using the k_a_ of 2.08 h^−1^ (hamster) and 1.54 h^−1^ (rat) [43]. The predicted human PK parameters of artesunate and dihydroartemisinin were 0.38 h^−1^ for k_a_, 0.01 L (=0.0002 L/kg) for V_ss_/F, 14.69 L/h (=0.21 L/h/kg) for CL/F, 25.04 L (=0.36 L/kg) for V_m_/(F·F_m_), and 108.50 L/h (1.55 L/h/kg) for CL_m_/(F·F_m_) in 70 kg human. The coefficient of determination (r^2^) was estimated to be 0.8230 for k_a_, 0.9931 for V_ss_/F, 0.0254 for CL/F, 0.9352 for V_m_/(F·F_m_), and 0.5105 for CL_m_/(F·F_m_), respectively. Figure 4. shows the exponents, coefficients, and correlation coefficients for simple allometric scaling to predict human PK parameters of artesunate and dihydroartemisinin.

## 3. Discussion

Reported PD data for drug repurposing. We have previously evaluated the PKs of pyronaridine and artesunate in hamsters to support the correlation between in vivo exposures and in vitro antiviral activity [37]. However, only C_avg_ and AUC were calculated for assessing in vivo exposures, and disposition parameters (e.g., volume of distribution and clearance) were not estimated. This study additionally evaluated the PKs of pyronaridine and artesunate via NCA and PK modeling to describe interspecies PK differences in animal species. The PK parameters in hamsters, rats, and dogs were extrapolated to the clinical PK parameters by the simple equation for allometric scaling. Since the dosage regimen for the approved pyronaridine (Pyramax^®^) is a single oral daily dose for three consecutive days, the multiple-dosing schedules were set the same as the approved drug. For example, only the single-dose PKs of pyronaridine in dogs have been performed to evaluate inter-species differences. Thus, PK modeling was used to integrate the single- and multiple-dose PK data to predict the clinical PKs.

In the NCA result of pyronaridine (Table S1), the low- or high-doses were orally administered daily for three days to hamsters. The exposure parameters (C_avg_, AUC_τ_, and AUC_inf_) were all about 1.5 times higher in the high-dose (360 mg/kg) group than the low-dose (180 mg/kg) group. Even though the exposure parameters are shown as less than dose-proportional, the t_1/2_ (13.97–14.05 h) and MRT (20.15–20.27 h) were not changed as the dose increased. Rats and dogs showed similar t_1/2_ (about 37–42 h), whereas hamsters showed relatively short t_1/2_ (about 14 h). The PKs of pyronaridine in several animal species (rat, rabbit, dog, and monkey) have been reported. After intramuscular administration of pyronaridine to monkeys and rabbits, the T_max_ was reached within 0.75–1.5 h with a t_1/2_ of 48–72 h [44,45]. In rabbits, the range of T_max_ by intragastric (IG) and intramuscular (IM) administration of pyronaridine was 1.38–1.62 h and 0.75 h, respectively [44]. In addition, the t_1/2_ after IG, IM, and IV administration to rabbits were 56, 49, and 59 h, respectively [44,46]. Using the PK profile after the IV injection in rabbits, the V_ss_ and CL were estimated to be 29.0 ± 6.0 L/kg and 0.442 ± 0.131 L/h/kg, respectively [44]. The PK parameters estimated from IG and IV administration were described using the compartment PK model. The monkey PK data (n = 1) for pyronaridine showed a t_1/2_ of 64 h [45]. Intravenous injection of pyronaridine in rats and dogs showed an apparent terminal t_1/2_ of 48–96 h [1,47]. 

In the NCA results of artesunate and dihydroartemisinin (Table S2), the hamster showed a similar t_1/2_ (1.18–1.39 h for artesunate; 0.31–0.33 h for dihydroartemisinin) and MRT (1.70–2.00 h for artesunate; 0.44–0.47 for dihydroartemisinin) between the low-dose (60 mg/kg) and high-dose (120 mg/kg) groups. The AUC_τ_ of artesunate on days 1 and 3 were calculated to be 151.07 and 15.88 nmol∙hr/L for the low-dose group, and 298.16 and 44.53 nmol∙hr/L for the high-dose group in hamsters. The AUC_τ_ of dihydroartemisinin on days 1 and 3 were calculated to be 3698.50 and 614.17 nmol∙hr/L for the low-dose group, and 31,147.39 and 2860.22 nmol∙hr/L for the high-dose group in hamsters. In rats, the AUC_τ_ on day 1 and day 3 were calculated to be 506.74 ± 170.31 and 69.01 ± 39.58 nmol∙hr/L for artesunate, and 943.37 ± 646.81 and 79.46 ± 69.28 nmol∙hr/L for dihydroartemisinin. Due to the reduced AUC after repeat dosing of artesunate, the accumulation index of artesunate and dihydroartemisinin was less than 1 in hamsters and rats. The volumes of distribution and clearance of dihydroartemisinin could not be estimated since the dose of the metabolite or the fraction of drug metabolized (F_m_), which is used for calculating the PK parameters of the metabolite, is unknown. To describe the PKs of dihydroartemisinin, artesunate was assumed to be eliminated through the central compartment, where the F_m_ of artesunate was metabolized to dihydroartemisinin. However, it was impossible to estimate the actual value of the F_m_ and the V_d_ of the dihydroartemisinin simultaneously [48,49]. Thus, the apparent clearance [CL_m_/(F·F_m_)] and apparent volume of distribution [V_m_/(F·F_m_)] of dihydroartemisinin were estimated.

Several studies have reported the PKs of artesunate and dihydroartemisinin after administering oral or intravenous artesunate in rats. After oral administration of 100 mg/kg artesunate to rats, the t_1/2_ was 1.14 ± 0.22 h for artesunate and 0.90 ± 0.21 h for dihydroartemisinin [50]. The IV injection study of artesunate showed a t_1/2_ of 0.46 ± 0.06 h for artesunate and 0.65 ± 0.06 h for dihydroartemisinin [51]. Also, the range of reported MRTs in rats was 0.20–2.05 h for artesunate and 0.40–0.65 h for dihydroartemisinin, respectively [51,52,53]. The accumulation index was reported to be 0.37–1.11 for artesunate and 0.53–0.80 for dihydroartemisinin, respectively, from the repeat-dose studies of artesunate in rats [51,52,53].

The volume of distribution at steady-state (V_ss_) is a key PK parameter that explains the relationship between the amount of drug in the body and concentration in blood at equilibrium [54,55,56]. Also, estimating V_ss_ is important since it influences t_1/2_ and C_max_, which supports determining dosage regimens in clinical trials [57]. In this study, compartment PK modeling of pyronaridine and artesunate was conducted in animal species to estimate the PK parameters, including V_ss_. In all animal species, the CV% for most estimated PK parameters was within about 30% (Table 1), and the observed PK profiles were well described by model predictions (Figure 1 and Figure 2). In the case of artesunate and dihydroartemisinin, several studies have shown the auto-induction PKs and the plasma concentrations (or AUC) showed a remarkable decline after repeated dosing in our NCA results [51,53]. Chai et al. have examined the mechanism for the auto-induction phenomenon of artesunate and dihydroartemisinin by 5-day IV/PO PK studies in rats and dogs [58]. In Chai’s study, the hepatic and intestinal first-pass effects were investigated with the induction capacities of cytochrome P450 (CYP450), and the result showed an increased CYP450 expression in the intestine. It suggests declining blood concentrations after multiple oral doses are caused by the increased intestinal first-pass effect rather than the hepatic first-pass effect or systemic elimination. To accurately capture the tissue-specific auto-induction and time-dependent PKs, future development of a physiologically-based pharmacokinetic (PBPK) model should be conducted with the auto-induction mechanism of an intestine compartment. The other issue in the NCA results is that the volume of distribution and the clearance for the metabolite could not be estimated since the administered dose of the metabolite or the parent drug-to-metabolite ratio are unknown. Thus, parent-metabolite PK modeling with auto-induction elimination was conducted to capture declining plasma concentrations and estimate the volume of distribution and clearance for dihydroartemisinin. The model structure and parameters for the parent-metabolite model and auto-induction elimination were modified from several pieces of literature [48,59,60].

Allometric scaling is the most common approach for interspecies scaling to describe the relationship of PK parameters between animal species and humans [56,61]. The major assumption for allometric scaling is that PK parameters are related to body weight [62]. In the current study, the estimated PK parameters of k_a_, CL/F, and V_ss_/F in animal species were extrapolated into those of humans using the simple allometric equation. In the case of first-order rate constants (units of reciprocal time; h^−1^), the power exponent of −0.25 is generally used [43]. Among 40 drugs, the typical range for simple allometric exponents is 0.35 to 1.39 for clearances [63,64]. Also, the exponent for all types of volume distribution was around 1 and generally ranged between 0.8 and 1.1 [64,65]. The allometric exponents (*b*) of pyronaridine for k_a_, V_ss_/F, and CL/F were estimated to be −0.1928, 0.8220, and 0.7296, respectively (Figure 3). The coefficient of determination (r^2^) for the k_a_ (0.2103) was relatively lower than the V_ss_/F (0.9564) and CL/F (0.9967). It might be since the extrapolation of ka with body weight was less evident than that of volumes of distribution and clearances [66]. The allometric exponents of the parent drug (artesunate) and the metabolite (dihydroartemisinin) were estimated to be −1.4725 for the V_ss_/F, −0.1547 for the CL/F, 1.2933 for the V_m_/(F·F_m_), and 0.2509 for the CL_m_/(F·F_m_) (Figure 4). Since the k_a_ in dog was unavailable, the allometric exponent for ka was fixed to be −0.25, which is the general exponent for first-order rate constants [43]. Unlike the typical reported values, the parent drug (artesunate) showed negative values of simple allometric exponents for the volume of distribution (−1.4725) and clearance (−0.1547), and only the metabolite (dihydroartemisinin) showed positive values for those parameters. These values suggest the volume of distribution and the clearance of artesunate decreased as the body weight increased, while those parameters of dihydroartemisinin increased as the body weight increased.

Several studies conducted population PK modeling of pyronaridine in healthy and malaria-infected adults, and the PKs of pyronaridine were well-fitted by a two-compartment model in both populations [67,68]. Also, artesunate disposition was reported to be best described by a two-compartment model in humans, capturing a rapid initial distribution phase [69]. The disposition pharmacokinetics of dihydroartemisinin were best described by a one-compartment model with no benefit of an additional peripheral compartment in humans [70,71]. All reported PK models followed first-order absorption and elimination kinetics for pyronaridine, artesunate, and dihydroartemisinin, which are the same in this study. Hence, the two-compartment model in dogs for pyronaridine and the combined one- and two-compartment model in rats for artesunate/dihydroartemisinin best described the human PKs and disposition.

The predicted human PK parameters were compared with the reported ones in the literature. Two studies have evaluated the human PKs of pyronaridine via compartment PK modeling. In one published Ph.D. dissertation, the k_a_ of 0.87 h^−1^, V_ss_/F of 7532 L, and CL/F of 20.08 L/h were estimated using the two-compartment population PK model in healthy adults [67]. The other literature performed population PK modeling by pooled analysis in malaria-infected pediatric patients younger than 16 years of age, and PK parameters were estimated to be 0.76 h^−1^ for k_a_, 5460 L for V_ss_/F, and 15.71 L/h for CL/F [68]. The predicted k_a_ (=1.44 h^−1^) and CL/F (=28.25 L/h) in this study were within 2-fold compared with the literature, while the predicted V_ss_/F showed about 3- to 4-fold lower than the observed values in humans. Also, several studies have evaluated the pharmacokinetics of artesunate and dihydroartemisinin following the administration of artesunate in humans. The reported k_a_ for the oral and intrarectal routes was 0.2–3.9 h^−1^ [72,73]. The range of the reported V_ss_ of artesunate was 0.09–15.2 L/kg for the IV route and 2.07–19.67 L/kg for the extravascular (PO and IM) routes [73,74,75,76]. The CL of artesunate was reported to be 1.16–64 L/h/kg for the IV route and 2.4–60 L/h/kg for the extravascular route, respectively [73,77,78]. The V_ss_ of dihydroartemisinin was reported to be 0.75–2.40 and 1.20–6.34 L/kg following the IV and extravascular (oral, intramuscular, and intrarectal) administration of artesunate, respectively [73,75]. The reported range of the clearance of dihydroartemisinin was 0.48–5.6 and 0.73–3.17 L/h/kg for the IV and the extravascular dosing of artesunate, respectively [72,73,74,75,76,78,79,80,81,82,83]. In this study, the predicted human PK parameters of artesunate and dihydroartemisinin from allometric scaling were 0.38 h^−1^ for k_a_, 0.0002 L/kg for V_ss_/F, 0.21 L/h/kg for CL/F, 0.36 L/kg for V_m_/(F·F_m_), and 1.55 L/h/kg for CL_m_/(F·F_m_), respectively. Both the predicted k_a_ of artesunate and the predicted CL of dihydroartemisinin were within the reported range of human PK parameters. However, the predicted CL of artesunate and V_ss_ of artesunate and dihydroartemisinin were more than two times lower than the reported values. This large difference between the actual CL and predicted CL of artesunate in humans might be due to the CL of artesunate being irrelevant to the body weight and showing a very low coefficient of determination (r^2^) of 0.0254. The unbound fraction of the drug can influence the V_ss_ in the blood (f_u,blood_), the unbound fraction of the drug in tissue (f_u,tissue_), and the tissue-to-blood partition coefficient (K_p_) [83]. A relationship between V_ss_ and blood–tissue volumes/protein binding/tissue partitioning is described as follows [83,84,85,86]:

$$V_{ss}=V_{b}+V_{t}\times \frac{f_{u,blood}}{f_{u,tissue}}$$

$$V_{ss}=V_{b}+\sum_{i}^{n}V_{t,i}\times K_{p,i}$$

where the V_b_ is the blood volume and the V_t_ is the sum of all tissue volumes. In this study, the V_ss_ in animal species were estimated by assuming that the f_u,blood_ and f_u,tissue_ are equal (V_ss_ = V_b_ + V_t_), and the human V_ss_ was predicted with more than a 2-fold difference between the predicted and actual measured value. This large difference in the V_ss_ might be due to the difference between the unbound fraction of the drug in blood (f_u,blood_) and the unbound fraction of the drug in tissue (f_u,tissue_). If the unbound fraction in tissue is more than two times lower than that in blood, the tissue binding fraction, or partitioning, is more than two times higher. It might result in a higher value of the actual V_ss_ than the predicted V_ss_. In the EMA report for pyronaridine, the f_u,blood_ has been reported to be in the range of 4% to 8% in rats, rabbits, dogs, and humans, while tissue concentrations were ten times higher than blood concentrations with a potential tissue accumulation in rats [5]. Artesunate was extensively distributed in the intestines, urinary bladder, bile duct, and small and large intestinal contents, with a K_p_ of 3.08–28.86 following IV injection to rats and showed similar protein binding rates of 73–86% between rats and humans [87,88]. Dihydroartemisinin also showed wide tissue distribution for 192 h after IV injection to rats with the K_p_ of 5.04–226.37 in 17 tissues and showed high binding rates (76–82%) with both rat and human plasma proteins [89]. Based on the evidence for the extensive tissue distribution of pyronaridine, artesunate, and dihydroartemisinin, the difference between the predicted and actual V_ss_ in humans could be derived from the higher tissue binding (f_u,blood_/f_u,tissue_ > 1) and higher tissue partitioning (K_p_ > 1). Despite this limitation, the inter-species scaling of other PK parameters was well described by the simple allometric equation, and it might be a useful tool for predicting clinical PK parameters. In the future, evaluating the tissue distribution of pyronaridine and artesunate could be combined with the PK parameter estimates, and it could allow a more comprehensive understanding of the prediction of clinical PK parameters.

International organizations have recently suggested alternative approaches, such as cell-based testing and computer modeling, to minimize and eliminate unnecessary animal testing [90]. Even though the FDA encourages and accepts scientifically valid alternatives to animal testing, validated alternatives are yet to be available for many medical products [91]. Although the use of animal models for pre-clinical testing has been minimized in some European countries and other regions, they have provided critical insights into drug pharmacokinetics, informing subsequent human studies. The choice of animal models was driven by the need to evaluate comprehensive in vivo ADME data accurately, which remains challenging to predict and validate fully through in silico or in vitro methods alone. Moreover, animal models can aid researchers in conducting sophisticated in silico or in vitro modeling by incorporating fundamental data from animal experiments. This research aims to provide a balanced approach, utilizing traditional animal PK experiments and modeling methods to understand comprehensive PK data. The findings from our study can be valuable for researchers where animal models are acceptable, and they can be utilized as in vivo validation data for future in silico/in vitro modeling.

## 4. Materials and Methods

### 4.1. Chemicals and Reagents

Pyronaridine tetraphosphate (CAS No. 76748-86-2), artesunate (CAS No. 88495-63-0), and dihydroartemisinin (71939-50-9) were provided by Shin Poong Pharm. Co., Ltd. (Seoul, Republic of Korea). Amodiaquine (CAS No. 6398-98-7), sodium phosphate tribasic dodecahydrate buffer (CAS No. 10101-89-0), diethyl ether (CAS No. 60-29-7), ortho-phosphoric acid (CAS No. 7664-38-2), formic acid (CAS No. 64-18-6), ammonium acetate (CAS No. 631-61-8), and trifluoroacetic acid (TFA; CAS No. 76-05-1) were purchased from Sigma-Aldrich (St Louis, MO, USA). Acetonitrile (CAS No. 75-05-8), methanol (CAS No. 67-56-1), methyl-tert-butyl ether (CAS No. 1634-04-4), and water (CAS No. 7732-18-5) were purchased from J.T. Baker (Phillipsburg, DE, USA). All other chemicals and reagents were HPLC or analytical grade. 

### 4.2. LC-MS/MS Conditions

**Pyronaridine** Liquid chromatography was conducted on the Agilent 1290 Infinity II LC System (Agilent Technologies Inc., Santa Clara, CA, USA) coupled to a 6490 Triple Quad Mass Spectrometer (Agilent Technologies Inc., Santa Clara, CA, USA). A Synergi Max-RP column (2.0 × 75 mm, 4 μm particle size, Phenomenex, Torrance, CA, USA) was used at a temperature of 25 °C. The mobile phase consisted of 0.04% TFA in water (mobile phase A) and methanol:acetonitrile = 3:1 (*v*/*v*) (mobile phase B) with a flow rate of 0.2 mL/min. A gradient elution was used for the chromatographic separation of pyronaridine as follows: 0.0–1.5 min, 10% B; 1.5–1.6 min, 10–60% B; 1.6–4.0 min, 60% B; 4.0–4.1 min, 60–10% B; 4.1–5.0 min, 10% B. The multiple reaction monitoring was set with a positive atmospheric pressure chemical ionization (APCI+) mode. The MRM transitions of 518.2 > 447.1 and 356.2 > 283.0 were used for pyronaridine and amodiaquine (internal standard, IS). The collision energy of 17 and 21 eV was used for pyronaridine and amodiaquine, respectively. 

**Artesunate and dihydroartemisinin** Liquid chromatography was conducted on the Shimadzu Nexera-X2 series (Shimadzu Corp., Tokyo, Japan) coupled with an LCMS-8040 (Shimadzu Corp., Tokyo, Japan). An Inertsil ODS column (2.1 × 100 mm, 5 μm particle size; GL Sciences, Tokyo, Japan) was used at a temperature of 25 °C. The mobile phase consisted of 10 mM ammonium acetate (mobile phase A) and acetonitrile (mobile phase B), with a 0.2 mL/min flow rate. An isocratic elution (mobile phase A:B = 10:90, *v*/*v*) was used for the chromatographic separation. The multiple reaction monitoring was set with a positive electrospray ionization (ESI+) mode. The MRM transitions were 402.05 > 267.10 for artesunate, 302.00 > 267.05 for dihydroartemisinin, and 299.95 > 283.15 for artemisinin (IS), respectively. The collision energies of 11, 9, and 7 eV were used for artesunate, dihydroartemisinin, and artemisinin, respectively. 

### 4.3. Sample Preparation

**Pyronaridine** 50 μL of rat or dog blood sample was mixed with 10 μL of the IS solution (1 μg/mL of amodiaquine in 50% methanol). An amount of 125 μL of 0.5 M sodium phosphate tribasic dodecahydrate buffer (pH adjusted to 10.3 with 85% ortho-phosphoric acid) and 500 μL of diethyl ether were added to the mixed sample, vortexed for 5 min, and centrifuged at 21,130× *g* for 5 min. Then, 300 μL of supernatant was collected and evaporated using nitrogen at room temperature. The dried residue was reconstituted with 100 μL of mobile phase (0.04% TFA:methanol:acetonitrile = 40:45:15, *v*/*v*/*v*) and vortexed for 5 min before centrifugation at 21,130× *g* for 5 min. Five μL of aliquot was injected into the UPLC-MS/MS system. 

**Artesunate and dihydroartemisinin** 50 μL of hamster or rat plasma were mixed with 10 μL of the IS solution (5 μg/mL of artemisinin in 50% methanol). An amount of 300 μL of acetonitrile was added to the mixed sample, vortexed for 5 min, and centrifuged at 21,130× *g* for 5 min. Then, 325 μL of supernatant was collected and evaporated using nitrogen at room temperature. The dried residue was reconstituted with 75 μL of mobile phase and vortexed for 5 min before centrifugation at 21,130× *g* for 5 min. Five μL of aliquot was injected into the UPLC-MS/MS system.

### 4.4. Pharmacokinetic Study Design

**Rat** Eleven male Sprague-Dawley rats were obtained from Orient Bio (Seongnam-si, Korea), and the mean body weight of the rats was 205.2 ± 7.3 g. All rats were maintained on a 12 h light/12 h dark cycle at a temperature of 23 ± 3 °C, relative humidity of 50 ± 20%, ventilation with 10–15 air changes/hour, and 150–200 lux of light. The animal study has received approval for research ethics from the Institutional Animal Care and Use Committee (IACUC, protocol number: IACUC220095) of CHA University. All rats were randomly divided into three groups, and each group received a single or multiple (once a day for three days) oral dose of 60/20 mg/kg pyronaridine tetraphosphate/artesunate. Blood samples (0.3 mL) were taken from the jugular vein into heparinized tubes and stored at −80 °C until sample analysis.

**Dog** Nine male beagle dogs were obtained from SaeronBio (Uiwang-si, Korea), and the mean body weight of the dogs was 9.5 ± 0.3 kg. All dogs were maintained on a 12 h light/12 h dark cycle at a temperature of 23 ± 3 °C, relative humidity of 55 ± 15%, ventilation with 10–20 air changes/hour, and 150–300 lux of light. The animal study has received approval for research ethics from the IACUC (protocol number: 21-KE-762) of KNOTUS Co., Ltd. (Incheon, Republic of Korea). All dogs received a single oral dose of 90 mg/head pyronaridine tetraphosphate (51.2 mg as pyronaridine). Blood samples (3 mL) were taken from the jugular vein into heparinized tubes and stored at −80 °C until sample analysis. 

**Hamster** The hamster PK data were adapted from the hamster PK study of pyronaridine and artesunate previously conducted in our published research [37]. One hundred eight male Golden hamsters were obtained from Janvier Labs (Le Genest-Saint-Isle, France), and the mean body weight of the hamsters was 102.01 ± 5.72 g. The detailed design for the hamster, rat, and dog PK study was summarized in Table 2.

### 4.5. Pharmacokinetic Evaluation

For the estimation of PK parameters for pyronaridine, non-compartmental analysis (NCA) was performed using WinNonlin software (version 8.4, Certara™, Princeton, NJ, USA). The elimination rate constant (k_e_) was estimated by linear regression analysis, and the elimination half-life (t_1/2_) was calculated by dividing ln 2 by k_e_. The time to maximum concentration (T_max_) and maximum blood concentration (C_max_) were obtained by visual observation of the blood concentration-time curve. The area under the blood concentration-time curve from zero to time t (AUC_t_) and the area under the first moment curve from zero to time t (AUMC_t_) were calculated by a linear trapezoidal method. The area under the blood concentration-time curve from zero to time infinity (AUC_inf_) was calculated as follows: AUC_t_ + C_last_/k_e_, where C_last_ is the last measurable concentration. The area under the first moment curve from zero to time infinity (AUMC_inf_) was calculated as follows: AUMC_t_ + (C_last_ × t_last_/k_e_) + (C_last_/k_e_^2^), where t_last_ is the time of last measurable concentration. The clearance (CL/F) was calculated as dose/AUC_inf_ in the single-dose group or dose/AUC_τ_ in the multiple-dose group. The mean residence time (MRT) was calculated as AUMC_inf_/AUC_inf_. The volume of distribution at steady-state (V_ss_/F) was calculated as follows: (Dose×AUMC_inf_)/AUC_inf_^2^ or CL/F × MRT. The average blood concentration at a steady state (C_avg_) in multiple doses was calculated by dividing the AUC_τ_ by the dosing interval (τ). The accumulation index was calculated as a ratio of the AUC_τ_ from day 3 to day 1. Log-linear regression and basic/log statistics (mean, SD, variance, CI, etc.) were used as statistical methods for the model-independent method. The statistically significant differences were analyzed using the Wilcoxon rank sum test of R software (version 2024.04.2).

### 4.6. Pharmacokinetic Modeling

**Figure 5:**
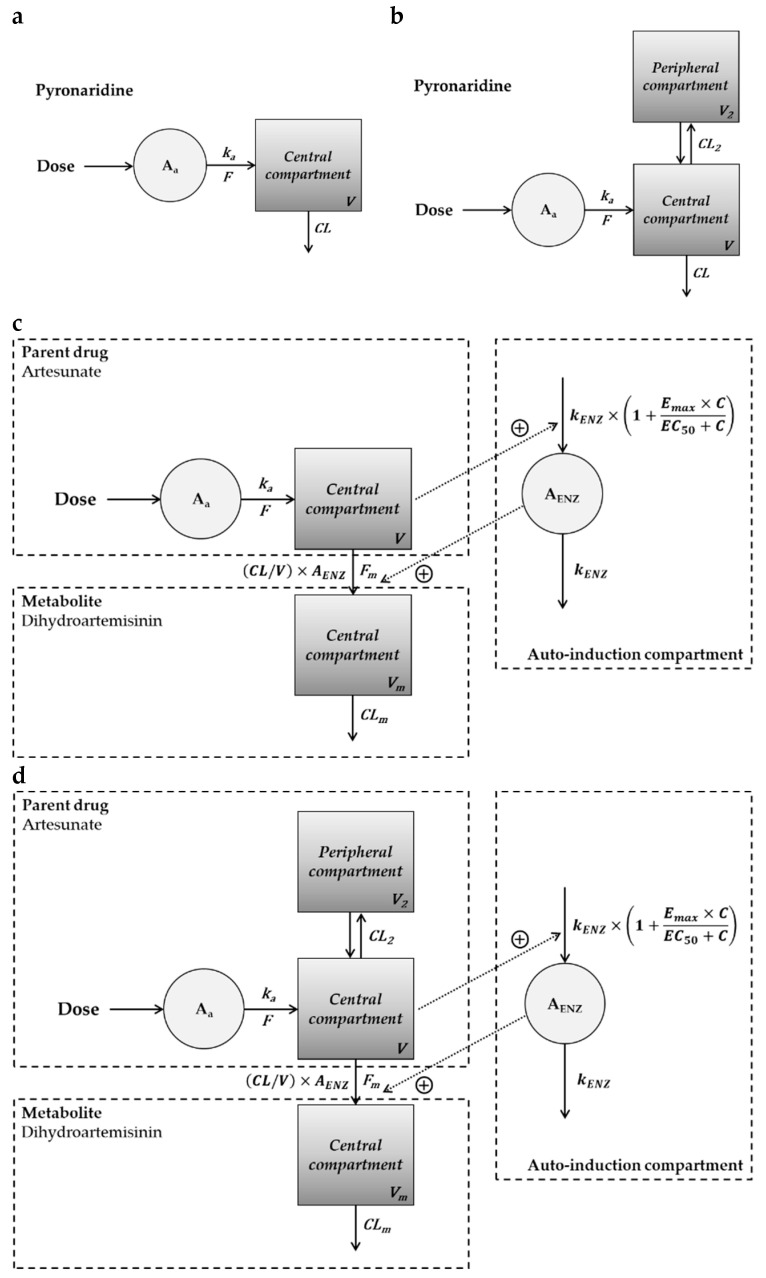
The developed PK models for pyronaridine and artesunate. (**a**) One-compartment model of pyronaridine in hamsters and rats. (**b**) Two-compartment model of pyronaridine in dogs. The parent-metabolite model with auto-induction of artesunate and dihydroartemisinin in (**c**) hamsters and (**d**) rats.

Compartment PK modeling was performed in three species (hamster, rat, and dog) to describe the time course of pyronaridine, artesunate, and dihydroartemisinin blood concentrations. The hamster PK data were collected from the literature we previously published [37]. The observed PK data in three species were fitted to a one- or two-compartment PK model using WinNonlin software (version 8.4, Certara™, Princeton, NJ, USA). The PK data were fitted using nonlinear mixed-effects (NLME) modeling with the first-order conditional estimation-extended least squares (FOCE-ELS) algorithm. The NLME model incorporated fixed effects (population parameters) and random effects (individual parameters). Also, a parent-metabolite model consisting of the one- or two-compartment PK model was developed to describe the PKs of artesunate and dihydroartemisinin. An auto-induction PK model was incorporated into the parent-metabolite model of artesunate and dihydroartemisinin since artemisinin-like drugs showed time-dependent PKs (decreasing plasma concentrations) after repeated oral administration by auto-induction metabolism [52,58]. First-order kinetics was assumed for the absorption, distribution, and elimination of pyronaridine, and a well-fitted model was chosen. The final models for pyronaridine, artesunate, and dihydroartemisinin are shown in Figure 5, and the differential equations for each model are provided in Table 3.

The PK parameters estimated from the NCA were used for initial estimates of the PK model parameters. Since hamster PK data were from a naïve pooled method, the mean blood concentrations were used for PK modeling. Population PK modeling was performed in rats and dogs using individual PK data. In the population modeling, the inter-individual variability (IIV) of PK parameters was modeled by exponential random effects as follows:

$$\theta_{i}=\theta_{TV}\times e^{\eta_{i}}$$

where *θ_TV_* is a population value of a PK parameter, *θ_i_* is the PK parameter for the *i*th individual, and *η_i_* is a random variable for the *i*th individual following a normal distribution with the mean of zero and the variance of ω^2^. The intra-individual variability (*ε*) was modeled by an additive error (pyronaridine) or multiplicative error (artesunate and dihydroartemisinin) as follows:

$$C_{obs}=C_{pred}+\varepsilon \;\text{(pyronaridine)}$$

$$C_{obs}=C_{pred}\times \left(1+\varepsilon \right)\;\text{(artesunate and dihydroartemisinin)}$$

where C_obs_ is observed concentrations and C_pred_ is predicted concentrations. The selection of the well-fitted model was guided by evaluating the precision of parameter estimates and diagnostic values, including the negative log-like (-2LL), Akaike information criterion (AIC), and Bayes information criterion (BIC). The developed model was evaluated using the visual inspection of PK profiles between observed and predicted concentrations and diagnostic plots, including the dependent variable (DV) versus (individual) predictions [(I)PRED], (conditional) weighted residuals [(C)WRES] versus population predictions (PRED), and (conditional) weighted residuals [(C)WRES] versus time after dose (TAD). A visual inspection of PK profiles between observed and predicted concentrations was also conducted.

### 4.7. Extrapolation into Clinical PK Parameters by Allometric Scaling

The human PK parameters were extrapolated from the hamster, rat, and dog PK parameters using allometric scaling. The hamster PK parameters were taken from our previous research [37]. The equation for simple allometric scaling was expressed as a power function following [92]:

$$Y=a\times W^{b}$$

where *Y* is the pharmacokinetic parameter of interest, *W* is the body weight, *a* is the coefficient of the allometric equation, and *b* is the exponent of the allometric equation. The above equation was log-transformed as follows:

log⁡Y=log⁡a+blog⁡W

where log *a* is the y-intercept and *b* is the slope. The PK parameters and actual body weights of three animal species (hamster, rat, and dog) were plotted using a log-log scale. Then, *a* and *b* were estimated by a curve fitting the equation to scale up the PK parameters of a 70 kg human. The k_a_, CL/F, and V_ss_/F were extrapolated to 70 kg men from animals.

## 5. Conclusions

The inter-species PK difference for pyronaridine and artesunate in hamsters, rats, and dogs was evaluated using NCA and compartment PK modeling in animal species. The simple allometric equation (*Y* = *a* × *W^b^*) well-described the inter-species difference of PK parameters according to the body weights. Given the reported human PKs of both drugs, dogs were well-correlated with humans for pyronaridine, and rats were well-correlated with humans for artesunate/dihydroartemisinin. The data from the study may support the rationale for extrapolating PK exposures to evaluate the clinical efficacy of pyronaridine and artesunate for new targets. These data could be used as a basis for drug repurposing of pyronaridine and artesunate, and the results from the study could support assessing clinical effectiveness based on predicted clinical PK exposures. Furthermore, in drug repurposing, these data could be applied to determine effective dosage regimens based on clinical PK-PD prediction for new indications in initial clinical trials.

## Figures and Tables

**Table 1:** PK parameter estimates of pyronaridine, artesunate, and dihydroartemisinin.

Parameter	Unit	Estimate	CV%	IIV * (%RSE)
**Pyronaridine**				
**Hamster**				
k_a_	1/h	11.62	30.21	-
V/F	L/kg	53.32	6.58	-
CL/F	L/h/kg	2.15	4.36	-
ε	-	0.36	7.95	-
**Rat**				
k_a_	1/h	1.67	8.84	0.02 (13.44)
V/F	L/kg	110.00	4.96	1.69 (16.25)
CL/F	L/h/kg	2.18	6.15	0.04 (28.17)
ε	-	0.34	4.13	-
**Dog**				
k_a_	1/h	2.46	22.43	0.46 (60.48)
V/F	L/kg	18.98	10.20	0.03 (142.17)
V_2_/F	L/kg	13.70	17.82	-
CL/F	L/h/kg	0.68	8.54	0.05 (48.35)
CL_2_/F	L/h/kg	1.30	28.88	-
ε	-	0.20	10.15	-
**Artesunate and dihydroartemisinin**				
**Hamster**				
k_a_	1/h	2.08	15.02	-
V/F	L/kg	1571.71	18.95	-
CL/F	L/h/kg	3555.94	9.60	-
V_m_/(F∙F_m_)	L/kg	0.02	11.70	-
CL_m_/(F∙F_m_)	L/h/kg	117.68	9.66	-
k_enz_	1/h	47.40	13.19	-
E_max_	-	7.06	10.70	-
EC_50_	nmol/L	0.42	13.37	-
ε (artesunate)	-	4.38	14.21	-
ε (dihydroartemisinin)	-	1033.76	12.12	-
**Rat**				
k_a_	1/h	1.54	24.85	
V/F	L/kg	33.03	14.81	
V_2_/F	L/kg	481.05	21.47	
CL/F	L/h/kg	13.33	14.61	
CL_2_/F	L/h/kg	82.69	13.45	0.18 (0.06)
V_m_/(F∙F_m_)	L/kg	0.16	25.23	
CL_m_/(F∙F_m_)	L/h/kg	237.25	34.09	1.13 (0.29)
k_enz_	1/h	1.04	28.48	1.32 (0.41)
E_max_	-	76.89	19.64	
EC_50_	nmol/L	0.49	42.60	
ε (artesunate)	-	35.19	18.81	-
ε (dihydroartemisinin)	-	194.67	25.47	-

* inter-individual variability (ω); IIVs in hamsters could not be estimated since the naïve-pooled method was used for PK modeling.

**Table 2:** Summary of pharmacokinetic study design for pyronaridine tetraphosphate/artesunate in hamsters, rats, and dogs.

Species	Group	N	Dosage Regimen (Pyronaridine/Artesunate)	Blood Sampling Time (Hour)	Ref.
Hamster	H1	60	180/60 mg/kg daily (for 3 days)	0, 0.08, 0.25, 0.5, 0.75, 1, 2, 4, 8, 12, 24, 47, 48.08, 48.25, 48.5, 48.75, 49, 50, 52, 56, 60, 72	[37]
Hamster	H2	48	360/120 mg/kg daily (for 3 days)	0, 0.08, 0.25, 0.5, 0.75, 1, 2, 4, 8, 12, 24, 47, 48.08, 48.25, 48.5, 48.75, 49, 50, 52, 56, 60, 72	[37]
Rat	R1	3	60/20 mg/kg once	0, (0.05, 0.08, 0.16, 0.33, 0.5, 0.75), 1, 2, 4, 8, 12, 24, 48, 96, 144	-
		3	60/20 mg/kg once	0, (0.05, 0.08, 0.16, 0.33, 0.5, 0.75), 1, 2, 4, 8, 12, 24, 48, 96, 144, 216	-
Rat	R2	5	60/20 mg/kg daily (for 3 days)	0, (0.05, 0.08, 0.16, 0.33, 0.5, 0.75), 1, 2, 4, 8, 12, 24, 48, (48.05, 48.17, 48.33, 48.5, 48.75), 49, 50, 52, 56, 60, 72, 96, 144, 192, 264	-
Dog	D1	9	90/- mg/head once	0, 0.5, 1, 2, 4, 6, 24, 72, 120	-

**Table 3:** The equations of the PK models for pyronaridine, artesunate, and dihydroartesunate.

**One-compartment model of pyronaridine in hamsters and rats**
$\frac{{dA}_{a}}{dt}=-k_{a}\cdot A_{a}$ $\frac{{dA}_{1}}{dt}=k_{a}\cdot A_{a}-C\cdot CL/F$ $C=A_{1}/V$
**Two-compartment model of pyronaridine in dogs**
$\frac{{dA}_{a}}{dt}=-k_{a}\cdot A_{a}$ $\frac{{dA}_{1}}{dt}=k_{a}\cdot A_{a}+C_{2}\cdot {CL}_{2}/F-C\cdot {CL}_{2}/F-C\cdot CL/F$ $\frac{{dA}_{2}}{dt}=C\cdot {CL}_{2}/F-C_{2}\cdot {CL}_{2}/F$ $C=A_{1}/V$ $C_{2}=A_{2}/V_{2}$
**The parent-metabolite model with auto-induction for artesunate and dihydroartemisinin in hamsters**
$\frac{{dA}_{a}}{dt}=-k_{a}\cdot A_{a}$ $\frac{{dA}_{1}}{dt}=k_{a}\cdot A_{a}-C\cdot CL/F\cdot A_{ENZ}$ $\frac{{dA}_{m}}{dt}=C\cdot CL/F\cdot A_{ENZ}-C_{m}\cdot {CL}_{m}/(F\cdot F_{m})$ $\frac{{dA}_{ENZ}}{dt}=k_{ENZ}\cdot \left(1+\frac{E_{max}\cdot C}{{EC}_{50}+C}\right)-k_{ENZ}\cdot A_{ENZ}$ $C=A_{1}/V$ $C_{m}=A_{m}/V_{m}$
**The parent-metabolite model with auto-induction for artesunate and dihydroartemisinin in rats**
$\frac{{dA}_{a}}{dt}=-k_{a}\cdot A_{a}$ $\frac{{dA}_{1}}{dt}=k_{a}\cdot A_{a}+C_{2}\cdot {CL}_{2}/F-C\cdot {CL}_{2}/F-C\cdot CL/F\cdot A_{ENZ}$ $\frac{{dA}_{2}}{dt}=C\cdot {CL}_{2}/F-C_{2}\cdot {CL}_{2}/F$ $\frac{{dA}_{m}}{dt}=C\cdot CL/F\cdot A_{ENZ}-C_{m}\cdot {CL}_{m}/(F\cdot F_{m})$ $\frac{{dA}_{ENZ}}{dt}=k_{ENZ}\cdot \left(1+\frac{E_{max}\cdot C}{{EC}_{50}+C}\right)-k_{ENZ}\cdot A_{ENZ}$ $C=A_{1}/V$ $C_{2}=A_{2}/V_{2}$ $C_{m}=A_{m}/V_{m}$

where A_a_, A_1_, A_2_, and A_m_ are drug amounts in the absorption, central, peripheral, and metabolite compartments, respectively. C, C_2_, and C_m_ are drug concentrations in the central, peripheral, and metabolite compartments, respectively. k_a_ is the absorption rate constant, and k_ENZ_ is the enzyme degradation rate (which also serves as a zero-order rate constant for enzyme production). CL, CL_2_, and CL_m_ are total, intercompartmental, and metabolite clearance, respectively. V, V_2_, and V_m_ are the volumes of distribution in the central, peripheral, and metabolite compartments, respectively. F is the oral bioavailability, and F_m_ is the metabolized fraction of the drug. E_max_ is the maximum induction in enzyme formation rate, and EC50 is the drug concentration that produces 50% of the E_max_.

## Data Availability

Data are contained within the article and Supplementary Materials.

## Supplementary Materials

The following supporting information can be downloaded at: https://www.mdpi.com/article/10.3390/ijms25136998/s1.
