# Supplementary material for: Inter-Species Pharmacokinetic Modeling and Scaling for Drug Repurposing of Pyronaridine and Artesunate
Source: Int J Mol Sci. 2024 Jun 26;25(13):6998. doi: 10.3390/ijms25136998 (PMC11241507; doi:10.3390/ijms25136998)
Supplement: Supplementary file 1 [file ijms-25-06998-s001.zip › ijms-3028386-supplementary.pdf]

Table S1. Estimated single- and multiple-dose PK parameters of pyronaridine tetraphosphate.

PK parameter	Unit	Single-dose			Multiple-dose	
		Rat (60 mg/kg)	Dog (90 mg/head)	Hamster[1] (180 mg/kg)	Hamster[1] (360 mg/kg)	Rat (60 mg/kg)
T_{max}	hr	3.83±3.43	1.67±1.09	-	-	-
C_{max}	µg/L	491.07±124.69	274.93±58.34	-	-	-
C_{avg}	µg/L	-	-	2378.52	3536.16	624.51±110.81
AUC_t	µg·hr/L	11737.12±779.55	7972.74±1839.02	-	-	-
$AUC_{Day\ 1}$	µg·hr/L	-	-	24134.35	51016.03	5075.92±1532.70
$AUC_{Day\ 3}$	µg·hr/L	-	-	57084.55	84867.91	14988.19±2659.49
AUC_{inf}	µg·hr/L	12306.56±931.78	8889.61±2357.39	-	-	-
$AUMC_{inf}$	µg·hr·hr /L	556235.60±130544.16	426762.07±210034.27	-	-	-
V_{ss}/F	L/kg	125.02±21.63	28.40±5.55	36.38	48.66	141.30±18.66
CL/F	L/hr/kg	2.79±0.21	0.64±0.13	1.80	2.41	2.33±0.36
$t_{1/2}$	hr	37.43±6.61	38.44±7.06	14.05	13.97	42.27±2.99
MRT	hr	45.01±8.60	46.19±11.47	20.27	20.15	60.99±4.31
Accumulation index	-	-	-	2.37	1.66	3.04±0.45

[1] Standard deviation could not be calculated since the naïve-pooled method was used to evaluate PKs in hamsters.

Table S2. Estimated single- and multiple-dose PK parameters of artesunate and dihydroartemisinin.

PK parameter	Unit	Single-dose	Multiple-dose		
		**Rat (20 mg/kg)**	**Hamster[1] (60 mg/kg)**	**Hamster[1] (120 mg/kg)**	**Rat (20 mg/kg)**
Artesunate					
T_{max}	hr	0.14±0.04	-	-	-
C_{max}	nmol/L	292.70±58.01	-	-	-
C_{avg}	nmol/L	-	0.66	1.86	2.88±1.65
AUC_t	nmol·hr/L	100.38±29.49	-	-	-
$AUC_{Day\ 1}$	nmol·hr/L	-	151.07	298.16	506.74±170.31
$AUC_{Day\ 3}$	nmol·hr/L	-	15.88	44.53	69.01±39.58
AUC_{inf}	nmol·hr/L	100.95±29.33	-	-	-
$AUMC_{inf}$	nmol·hr·hr/L	44.80±28.87	-	-	-
V_{ss}/F	L/kg	231.89±116.35	19674.11	11915.76	485.07±189.31
CL/F	L/hr/kg	547.08±132.41	9830.19	7009.38	1008.34±593.65
$t_{1/2}$	hr	0.17±0.07	1.39	1.18	0.43±0.25
MRT	hr	0.44±0.23	2.00	1.70	0.62±0.36
Accumulation index	-	-	0.11	0.15	0.13±0.06
Dihydroartemisinin					
T_{max}	hr	0.28±0.09	-	-	-
C_{max}	nmol/L	612.40±529.97	-	-	-
C_{avg}	nmol/L	-	25.59	119.18	3.31±2.89
AUC_t	nmol·hr/L	313.05±260.02	-	-	-
$AUC_{Day\ 1}$	nmol·hr/L	-	3698.50	31147.39	943.37±646.81
$AUC_{Day\ 3}$	nmol·hr/L	-	614.17	2860.22	79.46±69.28
AUC_{inf}	nmol·hr/L	318.97±257.25	-	-	-
$AUMC_{inf}$	nmol·hr·hr/L	156.30±111.94	-	-	-
$t_{1/2}$	hr	0.23±0.08	0.33	0.31	0.31±0.16
MRT	hr	0.52±0.11	0.47	0.44	0.45±0.23
Accumulation index	-	-	0.17	0.09	0.09±0.08

[1] Standard deviation could not be calculated since the naïve-pooled method was used to evaluate PKs in hamsters.

Table S3. The model development process for pyronaridine in hamsters, rats, and dogs.

Model	Description	-2LL	AIC	BIC	No. of parameters
**Hamster**					
1*	One-compartment model (low-dose group)	1578.82	1586.82	1597.20	4
	One-compartment model (high-dose group)	1679.76	1687.76	1698.22	4
2	Two-compartment model (low-dose group)	1594.41	1606.41	1621.98	6
	Two-compartment model (high-dose group)	1737.15	1749.15	1764.84	6
**Rats**					
1*	One-compartment model	1689.36	1697.36	1709.18	4
2	Two-compartment model	1683.13	1695.13	1712.87	6
**Dogs**					
1	One-compartment model	734.36	742.36	751.47	4
2*	Two-compartment model	720.10	732.10	745.76	6

* Final model

Table S4. The model development process for the parent-metabolite PK model of artesunate and dihydroartemisinin in hamsters and rats.

Model	Description	-2LL	AIC	BIC	No. of parameters
**Hamster (without auto-induction)**					
1	1-compt. + 1-compt. (low-dose group)	959.94	973.94	989.27	7
	1-compt. + 1-compt. (high-dose group)	655.76	669.76	683.94	7
2	1-compt. + 2-compt. (low-dose group)	959.93	977.93	997.63	9
	1-compt. + 2-compt. (high-dose group)	655.76	673.76	691.99	9
3	2-compt. + 1-compt. (low-dose group)	927.85	945.85	965.55	9
	2-compt. + 1-compt. (high-dose group)	620.12	638.12	656.35	9
4	2-compt. + 2-compt. (low-dose group)	963.17	985.17	1009.26	11
	2-compt. + 2-compt. (high-dose group)	618.13	640.13	662.41	11
**Hamster (with auto-induction)**					
5*	1-compt. + 1-compt. (low-dose group)	616.98	636.98	657.24	10
	1-compt. + 1-compt. (high-dose group)	857.94	877.94	899.84	10
6	1-compt. + 2-compt. (low-dose group)	625.82	649.82	674.13	12
	1-compt. + 2-compt. (high-dose group)	993.60	1017.60	1043.88	12
7	2-compt. + 1-compt. (low-dose group)	641.92	665.92	690.22	12
	2-compt. + 1-compt. (high-dose group)	947.98	971.98	998.26	12
8	2-compt. + 2-compt. (low-dose group)	617.71	645.71	674.07	14
	2-compt. + 2-compt. (high-dose group)	857.01	885.01	915.66	14
**Rats (without auto-induction)**					
1	1-compt. + 1-compt.	3027.71	3041.71	3065.95	7
2	1-compt. + 2-compt.	3027.71	3045.71	3076.88	9
3	2-compt. + 1-compt.	3027.71	3045.71	3076.88	9
4	2-compt. + 2-compt.	3027.71	3049.71	3087.81	11
**Rats (with auto-induction)**					
5	1-compt. + 1-compt.	3026.67	3046.67	3081.31	10
6	1-compt. + 2-compt.	2983.35	3007.35	3048.91	12
7*	2-compt. + 1-compt.	2936.07	2960.07	3001.64	12
8	2-compt. + 2-compt.	2978.67	3006.67	3055.16	14

* Final model

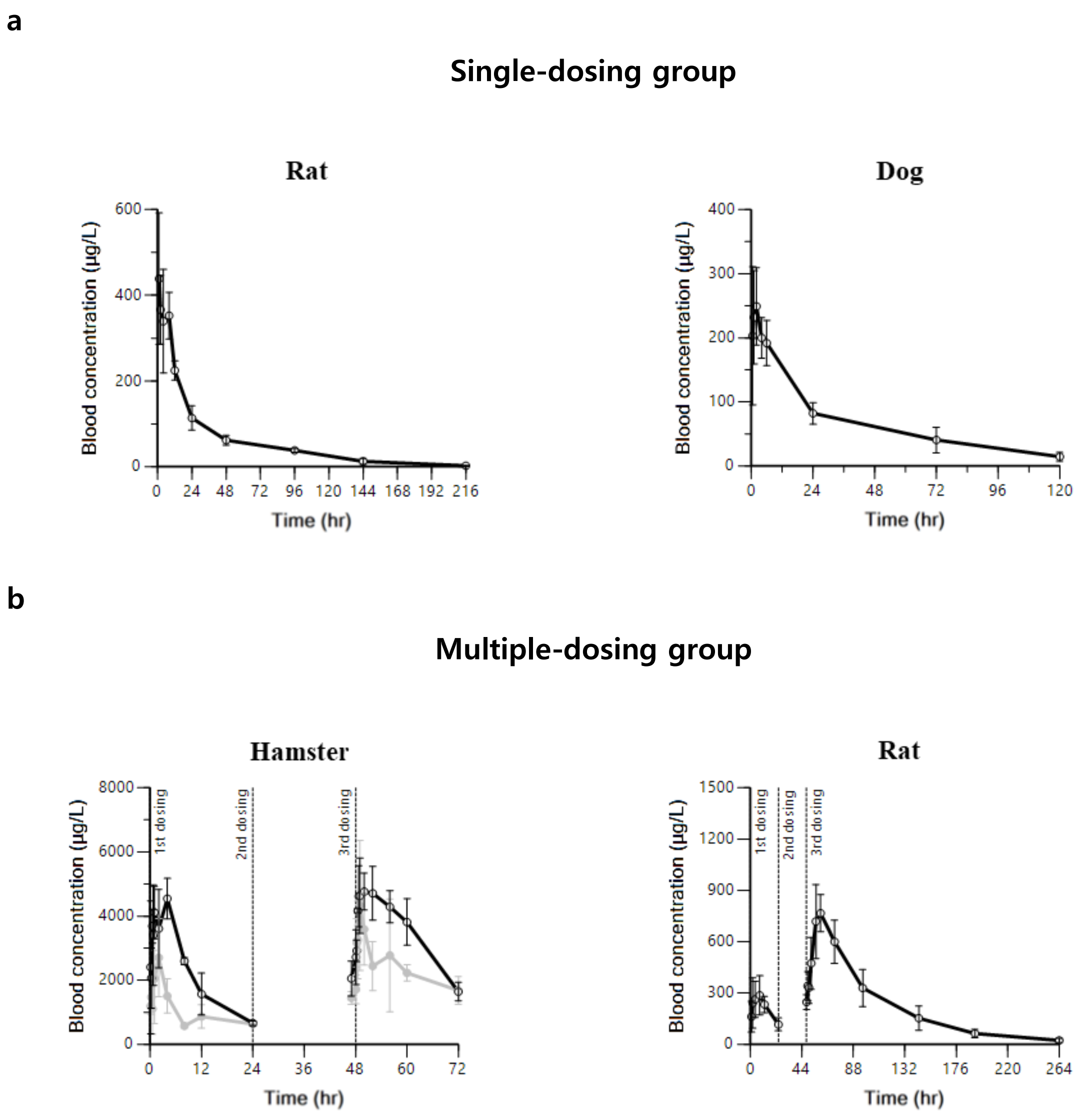

Figure S1. Pharmacokinetic profiles for single- or multiple-dose pyronaridine in hamsters, rats, and dogs. (a) Single-dose PK profile for rats and dogs. (b) Multiple-dose PK profiles for hamsters and rats. In the hamster PK profile, closed circles with gray lines represent the low-dose group, and open circles with black lines represent the high-dose group.

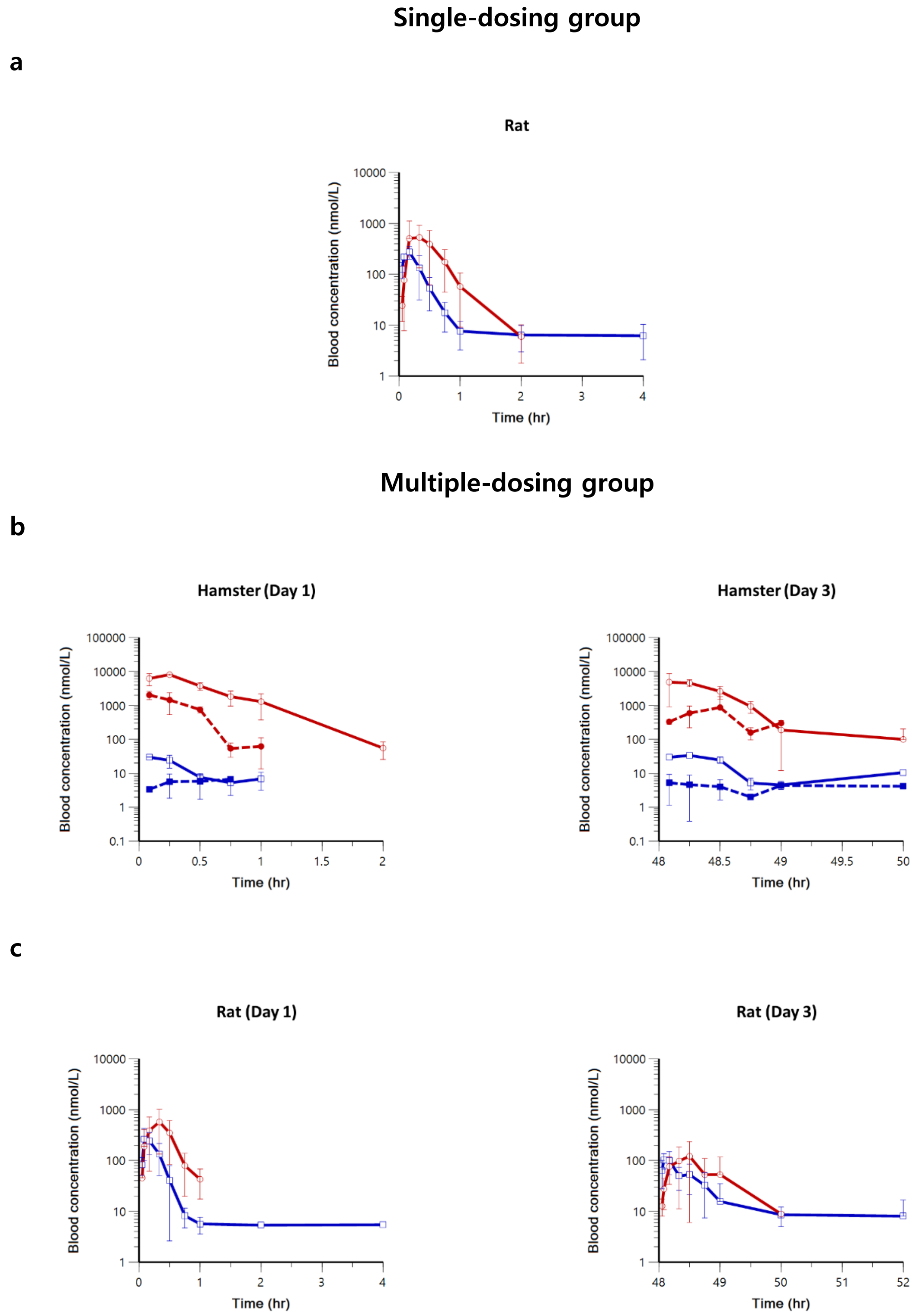

Figure S2. Pharmacokinetic profiles for single- or multiple-dose of artesunate in hamsters and rats. (a) Single-dose PK profile for rats. (b) Multiple-dose PK profile for hamsters. (c) Multiple-dose PK profile for rats. Blue squares with blue lines represent artesunate and red circles with red lines represent dihydroartemisinin, respectively. In the hamster PK profile, closed squares and circles with dashed lines represent the low-dose group, and open squares and circles with solid lines represent the high-dose group.

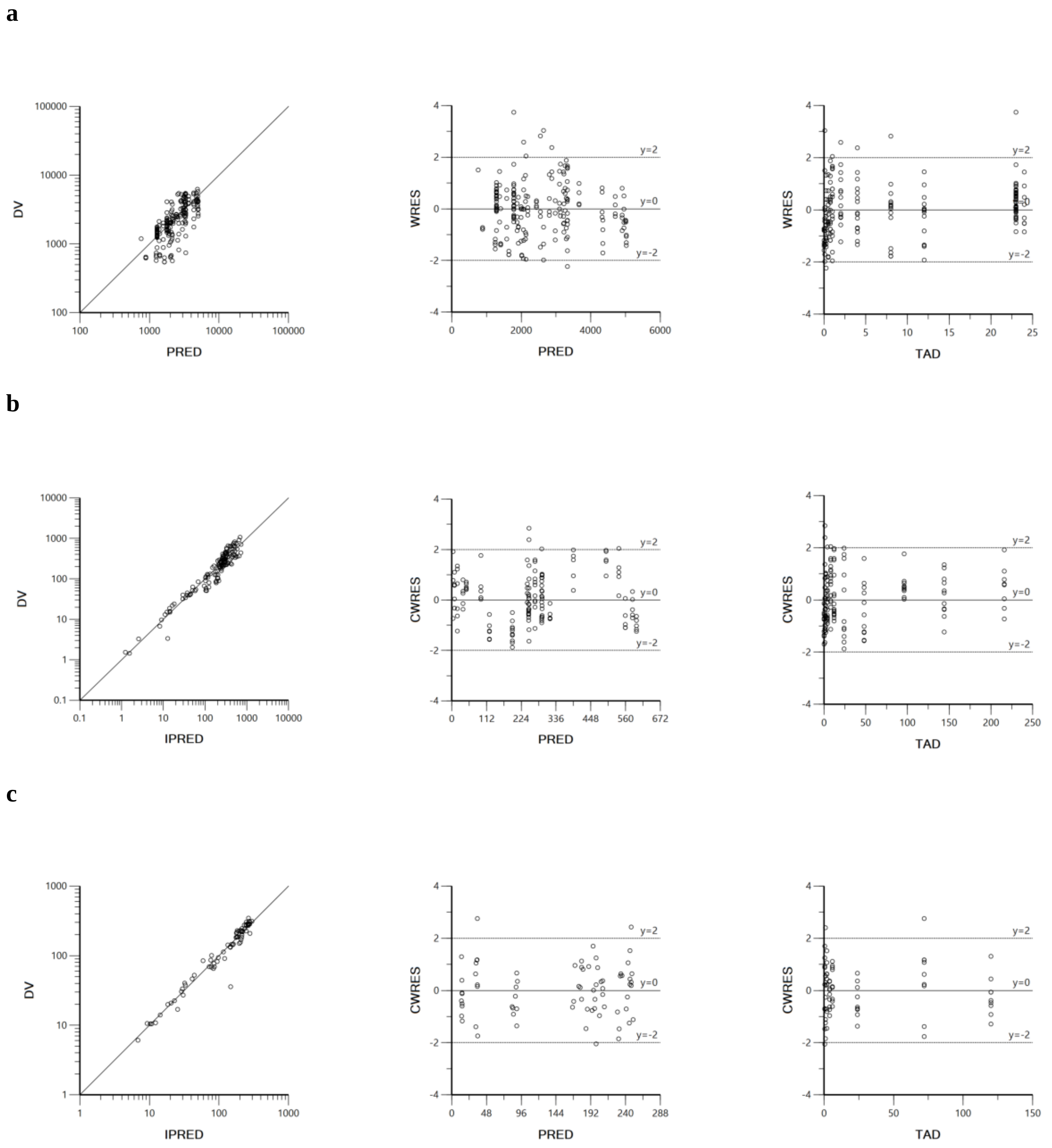

Figure S3. The diagnostic plots for the PK model of pyronaridine in (a) hamsters, (b) rats, and (c) dogs. The first column is DV vs (I)PRED plots, the second column is (C)WRES vs PRED plots, and the third column is (C)WRES vs TAD plots, respectively.

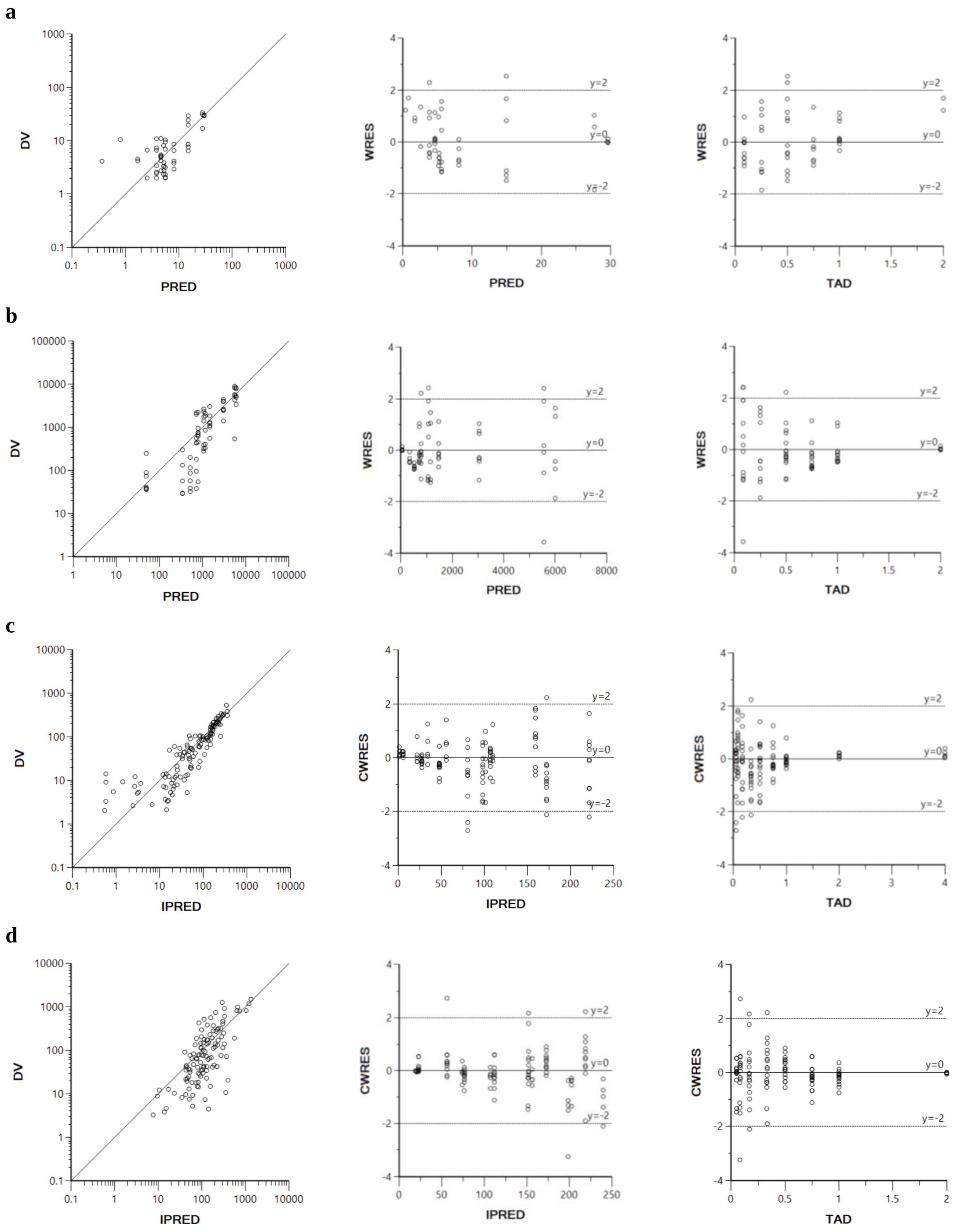

Figure S4. The diagnostic plots for the parent-metabolite PK model of artesunate and dihydroartemisinin. (a) artesunate and (b) dihydroartemisinin in hamsters. (c) artesunate and (d) dihydroartemisinin in rats. The first column is DV vs (I)PRED plots, the second column is (C)WRES vs PRED plots, and the third column is (C)WRES vs TAD plots, respectively.
